# Effectiveness of Chlorhexidine and Metronidazole Gels in the management of gingivitis. A clinical trial

**DOI:** 10.12669/pjms.37.5.4236

**Published:** 2021

**Authors:** Maryam Panhwar, Shazia Parveen Rajpar, Eisha Abrar, Montaser Alqutub, Tariq Abduljabbar

**Affiliations:** 1 Maryam Panhwar, Department of Community and Preventive Dentistry, Dow International Dental College, Karachi Pakistan; 2 Shazia Parveen Rajpar, Department of Community and Preventive Dentistry, Liaquat University of Medical Health Sciences Jamshoro; 3 Eisha Abrar, MDS Trainee Department of Operative Dentistry, Dow International Dental College, Karachi Pakistan; 4 Montaser Alqutub, Department of Periodontics and Community Dentistry, College of Dentistry, King Saud University, Riyadh, Saudi Arabia; 5 Tariq Abduljabbar Department of Prosthetic Dental Science, College of Dentistry, King Saud University; Research Chair for Biological Research in Dental Health , College of Dentistry, Riyadh 11545, Saudi Arabia

**Keywords:** Chlorohexidine, Metronidazole, Gingivitis, Randomized clinical trial

## Abstract

**Objectives:**

The purpose of the present study was to compare the topical application of chlorohexidine (CHX) and Metronidazole (MTZ) gels, individually and in combination in patients with gingivitis for up to 12 weeks follow-up.

**Methods:**

The clinical trial was conducted at Liaquat University of Medical Health Sciences (LUMHS) Jamshoro and Hyderabad, Institute of Dentistry from 1^st^ March 2019 to 1^st^ March 2020. Patients were selected based on inclusion criteria. Out of 125 screened patients, ninety-nine patients agreed to participate in the study. At the beginning of study all patients were assessed for gingival inflammation by using gingival index (GI) (Loe and silness, 1963). Scaling root planning (SRP) was performed in all patients. Subjects were randomly selected in three groups (n=33 each). In Group-A CHX gel was applied, Group-B Metronidazole gel was applied and the combination of two was applied to patients of Group-C. Patient follow up was done and gingival parameters were assessed at baseline, fourth week and twelve weeks. Apart from the clinical evaluation, a subjective evaluation was also undertaken. Significance level of 0.05 and a desired study power of at least 80% was estimated. Analysis of Variance (ANOVA) test for comparison was used within groups.

**Results:**

A significant improvement in gingival scores was noted in all groups from baseline. At 4 weeks CHX (1.25±0.21) MTZ (1.81±0.38) CHX+MTZ (1.29±0.34) compared to baseline CHX (2.77±0.24) MTZ (2.84±0.54) CHX+MTZ (2.74±0.31) demonstrated substantial improvement (p<0.001). However, gingival scores showed inclination at 12 weeks CHX (1.18±0.41) MTZ (1.21±0.48) CHX+MTZ (1.11±0.14) with no significant difference to week 4 (p>0.001).

**Conclusion:**

Local MTZ gel and MTZ+CHX gel showed effectiveness similar to CHX gel application adjunct to scaling and root planning in the treatment of gingivitis.

## INTRODUCTION

Gingivitis is characterized by inflammation of the gums, or gingiva. It commonly occurs due to film of plaque, bacteria accumulating on the tooth surface.^1^ Gingivitis is a non-destructive type of periodontal disease, but untreated gingivitis can progress to periodontitis. Epidemiological studies have estimated that the prevalence of adult gingivitis varies from approximately 50-100% in dentate patients.^2^ Existence of destructive types of microbes at sub gingival area results in development of periodontal disease.^3^ Anatomic factors of tooth and invasive nature of pathogens, resists mechanical instrumentation promoting bacterial colonization. Scaling and root planning (SRP) is the most commonly performed procedure for the treatment of periodontitis. Apart from conventional method use of different medicated agents can slow the progression of disease process.^4^

Metronidazole (MTZ) is a well-known antibiotic which is effective against various types of species including gram-negative rods and spirochetes.^5^ The use of antimicrobial therapy for treating gingivitis has persuaded interest in clinicians as the therapy is site specific, with reduced side effects and can be applied topically with better compliance and reduced systemic consequences.^6^ Along with numerous antimicrobial therapeutic agents, chlorhexidine (CHX) still remains ideal chemical with reasonable antiplaque efficacy and is considered one of the most effective topical antiseptics reported till date that has been successfully used for treating plaque-related gingivitis.^7^ However, till date there is no study present that compare the topical effects of CHX gel, MTZ gel and the combination of CHX and MTZ gel in gingivitis patients.

Various studies have been performed in evaluating the effects of systemic use of metronidazole only or along with the combination of SRP for the treatment of gingivitis. These types of studies displayed significant improvements based on microbiological and clinical assessments.^8^ This positive outcome has further reduced surgical needs for treatments of gingiva and supporting structures. Antimicrobial therapy in the form gel containing metronidazole, applied to specific marked site for pathogens resulted in providing higher concentration of drug.^9^ The present study was done to know the efficacy of MTZ and CHX gel form used alone and in combination in the treatment of gingivitis. Since, till date CHX is considered to be gold standard against plaque induced gingivitis it was hypothesized that CHX gel, MTZ gel and MTZ+CHX in combination can improve gingival parameters. Therefore, the purpose of the present study was to compare the topically application of CHX, MTZ alone and also in the combination of these two gels over a period of four and 12 weeks in subjects with gingivitis.

## METHODS

The study participants for the trial were opted from the patients attending Periodontology OPD at Liaquat University of Medical Health Sciences (LUMHS) Jamshoro and Hyderabad, Institute of Dentistry. The study was performed in accordance with the Declaration of Helsinki following the Consolidation Standards of Reporting Trials (CONSORT) Statement. The research ethical committee number was LUMHS/REC/779.

### Inclusion, Exclusion Criteria

A 12-week parallel arm RCT was performed. Complete procedure of the trial was explained to the subjects and a written consent was taken from all patients. The clinical trial was conducted from 1^st^ May 2019 to 1^st^ March 2020. Patients, aged between 12 to 40, having least 20 teeth’s present, bleeding on probing (BOP), probing depth ≤ 3 mm were included. Self-reported tobacco smokers, individuals using smokeless tobacco products, habitual alcohol users and patients with systemic diseases such as acquired immune deficiency, syndrome/HIV, diabetes mellitus, renal disorders and cardiovascular disorders were excluded.

### Study groups and randomization

Out of 125 screened patients, 99 patients agreed to participate in the study. At the beginning of study all patients were assessed for gingival inflammation by using the GI (Loe and silness,1963) (six sites per tooth) and after recording baseline scores every study participant were gone through full-mouth supra and sub gingival scaling with ultrasonic scalar irrigated with normal saline. Pre-or postoperative antibiotics were not prescribed in any group. All subjects were randomly selected by a computer-generated numbering sequence to one of the three groups (n=33 each)

### Group A - CHX

gel is applied on day 0 and for next 90 days.

### Group B – MTZ

applied on day 0 and for next 90 days.

### Group C – MTZ

in combination with CHX gel was applied for day 0 and next 90 days

**Fig.1:**
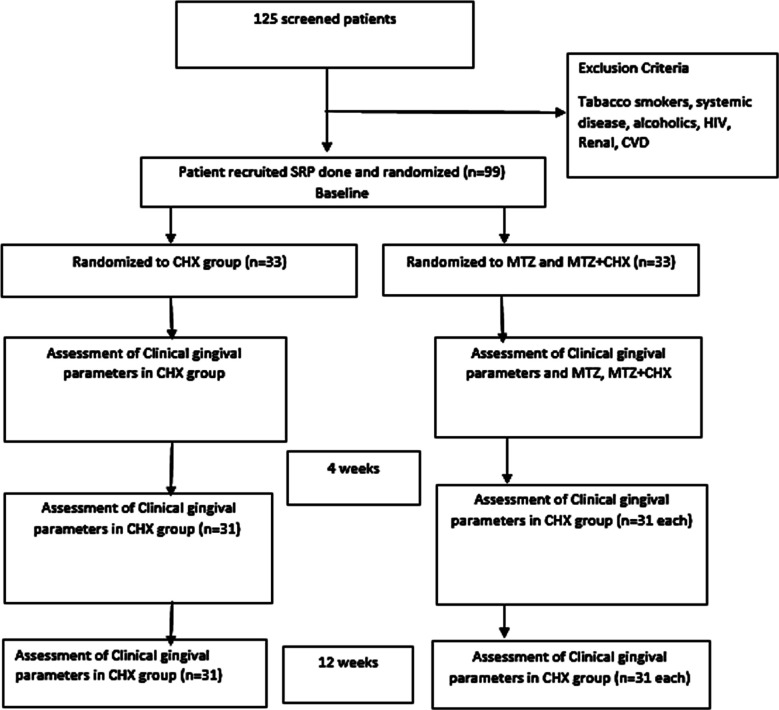
Flow Diagram.

All of our group participants were instructed to maintain proper oral hygiene (bass brushing technique) and to apply a pea-sized amount of gel gently with index finger to the gums after brushing for about two minutes twice a day. After application of different gels to all groups, further recording and scoring was done by principal investigator and other trained examiners at baseline (0 weeks), four weeks and 12 weeks. The overall kappa value for intra-examiner reliability was 0.86 (Fig.1).

### Qualitative Analysis

Apart from the clinical evaluation, a subjective evaluation was also undertaken at each visit using a questionnaire relating to the taste and flavor of the gels or any adverse effect experienced after use. To check for compliance, the participants were asked to return their assigned gel tubes so that the investigator could verify the amount of gel that was used

### Statistical Analysis

Normality of distribution of the variables was tested with Shapiro-Wilk tests. Data were expressed as means and standard deviations with mean percentages. Statistical Package for the Social Sciences (SPSS) 16.0 software was used. Power analysis was performed with a computer software (nQuery Advisor 5.0-Statistical Solutions, Saugus, Massachusetts). It was estimated that a sample size of at least 21 individuals per group were required. Significance level of 0.05 and a desired study power of at least 80% was estimated. Analysis of Variance (ANOVA) test for comparison was used within groups.

## RESULTS

Mean age of the patients in the present study was 25.09 ±7.198. The males and females were 58% and 42% respectively. All treatments groups were divided equally (n=33 each) and nine patients did not complete the study indicating a drop out of three patients from each group. None of the patients recruited in the study reported adverse outcomes with either of the three therapies used.

### Gingival Parameters:

A significant improvement in gingival scores was noted in all groups from baseline. At four weeks CHX (1.25±0.21) MTZ (1.81±0.38) CHX+MTZ (1.29±0.34) compared to baseline CHX (2.77±0.24) MTZ (2.84±0.54) CHX+MTZ (2.74±0.31) demonstrated substantial improvement (p<0.001). However, gingival scores showed inclination at 12 weeks CHX (1.18±0.41) MTZ (1.21±0.48) CHX+MTZ (1.11±0.14) with no significant difference to week 4 (p>0.001) (Table-I).

**Table-I:** Comparison of gingival parameters at baseline, 4 weeks and 12 weeks between experimental groups.

Interventional groups	Baseline Gingival Scores	4 weeks Gingival Scores	12 Weeks Gingival Scores
CHX	2.77±0.24 *	1.25±0.21	1.18±0.41
MTZ	2.84±0.54 *	1.81±0.38	1.21±0.48
MTZ+CHX	2.74±0.31 *	1.29±0.34	1.11±0.14

* Significant difference at different time points within the group at p<0.001.

## DISCUSSION

The present study was constructed on the hypothesis that CHX gel, MTZ gel and MTZ+CHX in combination can improve gingival parameters. All the three gels CHX, MTZ and MTZ+CHX when used improved gingival scores compared to baseline. However, gingival scores at 4 weeks and 12 weeks were statistically not significant. Based on the findings of the present study our hypothesis was accepted as all types of topical gel formation improved gingival scores.

Treatment of periodontal conditions is based on the removal of supra and sub gingival deposits of bacteria.^9^ This is accomplished by proper SRP. This non-surgical type of treatment is considered to be the cornerstone in treating all gingival and periodontal conditions. The approach is recommended by all dentists around world.^10^ The systemic use of antibiotics with SRP in periodontal related conditions has displayed valuable improvements in clinical results.^11^ But then we cannot ignore the side effects after using these antibiotics.^6^ To curtail the effects of systemic antibiotics, experts have developed new types of local drug delivery systems. Nature of these topical delivered antibiotic can be directed to periodontal pockets with a greater concentration, no or limited effect on oral microflora, with better absorption at target sites.^12^,^13^

In the present study CHX gel alone showed improvement in gingival scores from baseline at four and 12^th^ week. CHX has a wide spectrum of activity encompassing Gram positive and Gram-negative bacteria.^13^ CHX acts on the cell wall of bacterial structure resulting in lysis and destruction of bacteria.^14^,^15^ Work by Lander et al., investigated the clinical and biological impact of CHX gel and CHX solution on patients with gingivitis with no difference was found with gingival site treated with gel and solution.^16^ Discrepancy in results can be associated to study design, duration of study and concentration and formulation of CHX. However, Pannuti et al., in his study found CHX to be effective against gingivitis along with controlling inter dental bleeding. The outcome of the present study corelates with the findings of Pannuti et al.^17^

In the existing study 0.8% MTZ alone and in combination with CHX was used. Though the gingival scores improved from baseline to 4^th^ week but from 4^th^ to 12^th^ week gingival scores enhanced but this improvement was statistically insignificant. These results indicate that MTZ alone or in combination was efficacious to CHX. The findings of the present study were in accordance with work by Perinetti et al.^18^,^19^ The result also indicates that combination gel demonstrates an additive effect of both components. The effectiveness of MTZ to in periodontal conditions can be attributed to strong actions against anaerobes, limited unwanted effects of oral microflora and maximal absorption capacity.^12^,^20^ The present results indicate that MTZ applied topically compared to systemically diminishes antibiotics side effects.^19^,^21^

In the current study 21% of subjects reported an unpleasant taste and discoloration of teeth following the use of CHX gel. Three percent of those using the combination gel reported the same thing. The tooth staining is thought to be the result of a local precipitation reaction between tooth-bound CHX and chromogens found within foodstuffs and beverages.^22^

Single center study, absence of SRP group as control and minimal inhibitory concentration (MIC) of MTZ was not assessed and this can be counted as possible limitations of the study. Moreover, it is recommended to perform microbiological culture analysis in subjects after the use of MTZ and MTZ+CHX gel. In addition, long follow-up RCTs are required in order to evaluate the long-term effects of MTZ use in patients with gingivitis.

## CONCLUSION

Local Metronidazole gel and metronidazole and chlorhexidine gel showed effectiveness similar to CHX gel application adjunct to scaling and root planning in the treatment of gingivitis.

### Authors’ Contribution:

**MP and SPR:** Data collection, study design, manuscript writing, final manuscript approval.

**EA and MP:** Data collection, study design, manuscript drafting, data analysis, manuscript approval.

**MA and TAJ:** Data collection, manuscript approval and data interpretation.

**MA and TAJ:** Data collection, writing, editing and final manuscript approval.

**TAJ** is responsible and accountable for the accuracy or integrity of the work.
